# Carotenoids, total polyphenols and antioxidant activity of grapes (*Vitis vinifera*) cultivated in organic and conventional systems

**DOI:** 10.1186/1752-153X-6-66

**Published:** 2012-07-04

**Authors:** Claudiu-Ioan Bunea, Nastasia Pop, Anca Cristina Babeş, Cristian Matea, Francisc V Dulf, Andrea Bunea

**Affiliations:** 1 Department of Horticulture and Landscaping, University of Agricultural Sciences and Veterinary Medicine, Mănăştur 3-5, 400372, Cluj-Napoca, Romania; 2 Department of Biochemistry, University of Agricultural Sciences and Veterinary Medicine, Mănăştur 3-5, 400372, Cluj-Napoca, Romania

**Keywords:** Grapes-*Vitis vinifera*, Carotenoids, HPLC, Total polyphenols, Antioxidant activity, Organic farming

## Abstract

**Background:**

Organic agriculture involve plants which are cultivated without using synthetic pesticides, herbicides or fertilizers and promotes biodiversity, biological cycles and improve the product quality. The carotenoids, total polyphenols and the antioxidant activity from skins of some wine and table grapes cultivated in organic and conventional agriculture were studied.

**Results:**

The main carotenoids identified using high performance liquid chromatography were lutein and ß-carotene. Muscat Ottonel variety has the highest ß-carotene concentration 504.9 μg/kg for organic and 593.2 μg/kg for conventional grapes. For the organic farming, the total polyphenols content were in the range of 163.23 – 1341.37 mg GAE/kg fresh weight (FW) and 148.47 – 1231.38 mg GAE/kg FW for the conventional grapes. The highest ORAC values were obtained for blue-black variety Napoca in both farming system (43.5 ± 0.95 μmol TE/g organic; 40.4 ± 0.5 μmol TE/g conventional) and lowest for Aromat de Iaşi (16.8 ± 0.6 μmol TE/g organic; 14.7 ± 1.6 μmol TE/g conventional). Napoca variety showed also the highest antioxidant activity measured by DPPH method in both cultivated system.

**Conclusion:**

Nine grape varieties cultivated in organic and conventional systems were compared regarding the carotenoids, total polyphenols and antioxidant activity. The white grape varieties have a higher carotenoids content compared with the blue-black cultivars while the blue-black varieties contain higher TPC and exhibit higher antioxidant activity (except for Muscat Hamburg-ORAC). *Vitis vinifera* grape skins originating from wine or table grape varieties can be used as a potential source of natural antioxidants.

## Background

The grape berries are important since they are consumed as fruits, wine, juice or raisins and are largely cultivated for the wine industry. Grapes contain a wide range of chemical substances such as sugars, organic acids, mineral salts, vitamins, enzymes and also phytochemicals which are responsible for the sensory characteristics of wines [1] and for their health properties [2].

Antioxidant activities of grapes are due to the presence of antioxidant components such as flavonoids, phenolic acids, anthocyanins and carotenoids. Carotenoids play important roles in human nutrition through their provitamin A activity, but also by acting as antioxidants, for prevention of age-related macular degeneration or skin protection against UV radiation [3]. The antioxidant capacity of carotenoids was proved for pure compounds as well as for plant extracts and food [4,5]. Recent studies investigate the profile of carotenoids in some Italian grape varieties [6] and the effect of lightening and irrigation on carotenoids concentration [7,8].

Organic agriculture does not use synthetic pesticides and fertilizers [9] just ecological products during the cultivation. Higher concentration of bioactive compounds in plants grown in organic agriculture may be the results of the plant exposure to situation resulting from the absence of pesticides that leads to an increase of natural defense substances [10]. There are some studies concerning the influence of organic cultivation on the secondary metabolites in strawberries, yellow plumes and tomatoes [11,12]. It was shown that organic crop influenced the phenolic content and the antioxidant activity of white and purple grape juices [13]. Mulero et al. [14] studied the phenolic content and the antioxidant activity in Monastrell grapes obtained by organic and conventional agriculture. Vinković Vrček et al. [15] evaluate the antioxidant capacity, the polyphenols and metal content of conventionally and organically Croatian wines. The antioxidant activity of organically grown crops is reported to be higher [16] or there are no differences reported in the organic or conventional foods [17]. In eggplant cultivars grown with organic and conventional farming practices there were found no significant trend regarding the phenolic acids content [18]. The aim of this study was to compare the carotenoids, the total polyphenols content and the antioxidant activity from skins of some wine and table grapes cultivated in organic and conventional systems.

## Results and discussions

### Carotenoids

**Figure 1:**
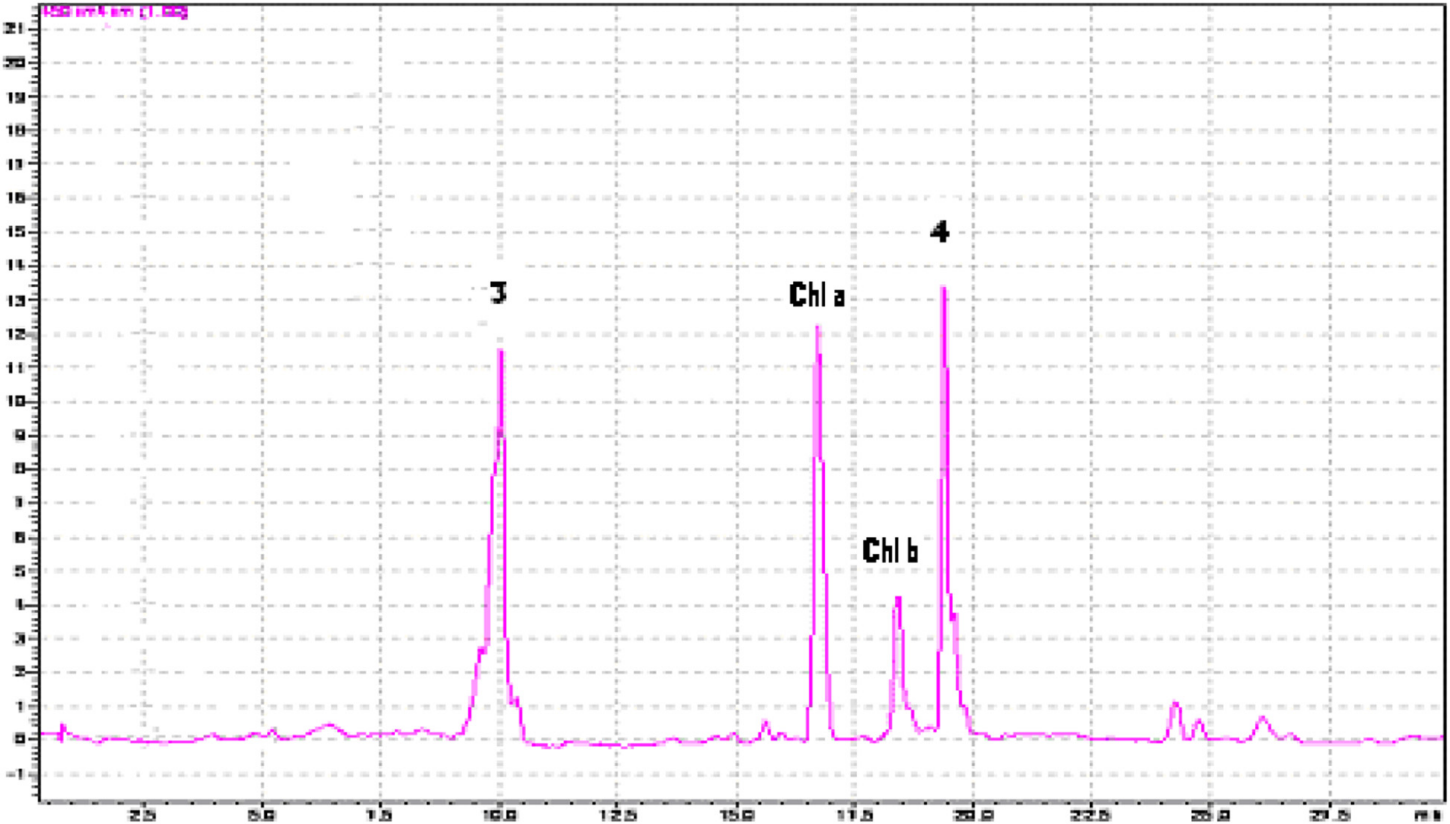
**HPLC-PDA chromatogram of carotenoids separated from fresh skin grapes (Muscat Ottonel variety-conventional).** The carotenoids identified are: (3) lutein and (4) b-carotene.

**Figure 2:**
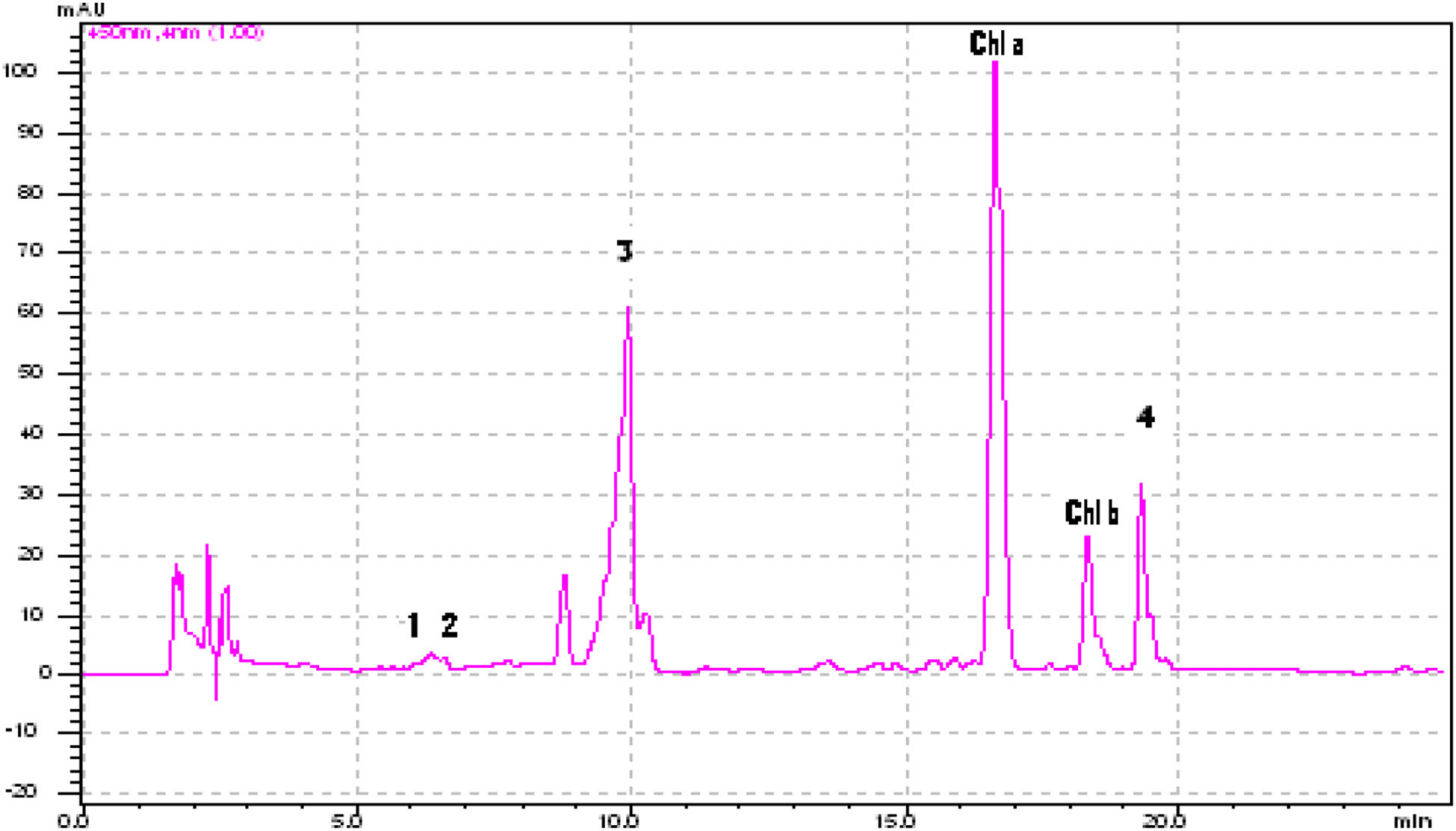
**HPLC-PDA chromatogram of carotenoids separated from fresh skin grapes (Muscat Hamburg variety-organic).** Four carotenoids were identified: (1) neoxanthin, (2) violaxanthin, (3) lutein and (4) ß-carotene.

The HPLC chromatograms for the carotenoids separated from the skin of Muscat Ottonel and Muscat Hamburg grapes are presented in Figure 1 and Figure 2. The main carotenoids identified were lutein and β-carotene. The most important carotenoids (85% from all the carotenoids present) identified in grapes are ß-carotene and lutein, the rest of them being neochrome, neoxanthin, violaxanthin, luteoxanthin, flavoxanthin, zeaxanthin and *cis-*isomers of lutein and ß-carotene [19]. Carotenoids are directly involved in grapes aroma because they can suffer degradation reactions followed by apparition of norisoprenoid compounds [20].

In Table 1 are presented the carotenoids concentration (ß-carotene and lutein) in grape skin extracts. When organic system was used it can be observed that the highest concentration of lutein was recorded in Fetească regală variety (855 μg/kg) followed at significant difference by Timpuriu de Cluj (799 μg/kg) and Muscat Ottonel (723 μg/kg) varieties, while the lowest concentration was obtained for Aromat de Iaşi (512 μg/kg). In conventional type of culture, Fetească regală (825 μg/kg) and Timpuriu de Cluj (803 μg/kg) has the highest lutein content. Regarding the ß-carotene content, Muscat Ottonel variety has the highest concentration (504.9 μg/kg-organic and 593.2 μg/kg-conventional, respectively) in both systems. The lowest ß-carotene content in organic system was found in Chasselas doré variety (273.5 μg/kg). In the conventional system, Chasselas doré and Riesling Italian varieties has statistically no differences between the values obtained for ß-carotene. The lowest ß-carotene values in conventional system were obtained for Napoca (229 μg/kg) and Aromat de Iaşi varieties (232 μg/kg).

**Table 1:** **The carotenoids concentration (μg/kg*)*in grape skin extract**

**Variety**	**Color**	**Organic**		**Conventional**	
		**ß-carotene**	**Lutein**	**ß-carotene**	**Lutein**
		**(μg/kg sample)**	**(μg/kg sample)**	**(μg/kg sample)**	**(μg/kg sample)**
Aromat de Iaşi	white	304.0 ± 49^de^	512.0 ± 69^g^	232.0 ± 46^e^	470.9 ± 46^e^
Traminer roz	white	331.9 ± 23^c^	551.0 ± 35^f^	356.8 ± 34^bc^	598.0 ± 53^d^
Riesling italian	white	365.2 ± 34^b^	632.0 ± 56^e^	264.5 ± 47^d^	709.0 ± 87^b^
Feteasca regală	white	328.3 ± 31^cd^	855.0 ± 66^a^	344.3 ± 32^bc^	825.0 ± 39^a^
Muscat Ottonel	white	504.9 ± 43^a^	723.0 ± 56^c^	593.2 ± 35^a^	602.0 ± 87^cd^
Timpuriu de Cluj	white	290.4 ± 14^ef^	799.0 ± 23^b^	334.1 ± 12^c^	803.0 ± 52^a^
Napoca	blue-black	369.0 ± 26^b^	701.0 ± 34^cd^	229.6 ± 18^e^	689.0 ± 19^b^
Chasselas doré	white	273.5 ± 23^f^	552.0 ± 56^f^	363.6 ± 45^b^	589.0 ± 21^d^
Muscat Hamburg	blue-black	302.1 ± 56^e^	685.0 ± 67^d^	265.0 ±32^d^	662.0 ± 12^bc^
LSD 0.05		25.77	31.27	22.86	51.57

Values are mean ± SD, n = 3; in each column, mean values with different letters are significantly different at P < 0.05.

There are relatively few studies regarding grape carotenoids composition. The quantitative carotenoids data obtained are in agreement with the literature data. Crupi et al. [21] studied the carotenoids concentration in some wine grapes and obtained values for lutein concentration in the range 130–682 μg/kg and for ß-caroten between 590–1370 μg/kg depending on the grape variety studied. Another study reported a lutein concentration between 218–1044 μg/kg and ß-carotene concentration of 168–910 μg/kg for different varieties [22]. The lutein to ß-carotene ratio in grapes, are highly variable depending on the viticulture region and on the variety analyzed. In some varieties (Sauvignon, Pinot Noir and Merlot) some authors found that lutein level was almost double compared to ß-carotene while in other varieties the concentration of this two carotenoids are similar or even higher for ß-carotene [23].

The profile and the amount of grape carotenoids could be influenced by several factors, such as: plant variety, climate conditions, stage of maturation, soil features [24].There are studies showing that soil irrigation has less influence on carotenoids profile compared with the type of soil and its water holding capacity. Soil with a low water holding capacity can lead to an increase of carotenoids concentration [25].

### Total polyphenols

In Table 2 is presented the total polyphenols content in grape skin extracts. The total polyphenols content (TPC) was determined using the Folin-Ciocâlteu method. Gallic acid was used as standard and the results (as gallic acid equivalents) were expressed as means ± standard deviation of triplicate analysis. When the organic system was applied the TPC values were in the range of 163.26 – 1341.37 mg GAE/kg fresh weight (FW) while for conventional cultivated grapes the TPC values were between 148.47 – 1231.38 mg GAE/kg FW. Among the grape variety cultivated in organic system, Napoca revealed the highest TPC as 1341.37 mg GAE/kg FW followed at significant difference by Muscat Hamburg (978.08 mg GAE/kg FW). The values obtained were higher compared with those obtain by Mulero et al. [14] for Monastrell variety (600 mg GAE/kg) and closer to the values obtained by Valls et al. [26]. Between grapes cultivated in conventional system, Napoca has the highest TPC (1231.38 mg GAE/kg) followed by Muscat Hamburg (953.04 mg GAE/kg) while the lowest value was found for Chasselas doré (148.47 mg GAE/kg). Regarding white conventional grape varieties, Fetească regală has the highest TPC content (575.71 mg GAE/kg FW) followed by Muscat Ottonel, Riesling italian, Traminer roz, Aromat de Iaşi and Timpuriu de Cluj. Significant differences were found in TPC for all the grape varieties analyzed (p < 0.05). Dani et al. [13] reported higher polyphenols content in red grape juices from *Vitis labrusca* compared with white ones. The range of total polyphenols in skin is lower than those reported by Lutz et al. [27] who analyzed four grape varieties and obtained values between 63.2-129 mg GAE/g. Junh et al. [28] and Katalinić et al. [29] also obtain higher values. The last one reported 875 mg GAE/kg (average) in seven white grape varieties grown in Dalmatia (Croatia). Previous studies reported lower total polyphenol values than those found in the present study [14,30]. This diversity of results can be due to the grape variety analyzed, growing conditions and/or the methods of extraction and analysis used [27].

**Table 2:** Total polyphenol concentration in grape skin extracts

**Variety**	**Colour**	**Organic**	**Conventional**
		**Total polyphenols content**	**Total polyphenols content**
		**(mg GAE/kg sample)**	**(mg GAE/kg sample)**
Aromat de Iaşi	white	220.38 ± 14.21^g^	228.84 ± 6.23^h^
Traminer roz	white	219.33 ± 4.02^g^	330.37 ± 2.36^g^
Riesling italian	white	423.43 ± 10.36^e^	436.12 ± 11.02^e^
Feteasca regală	white	579.94 ± 11.12^d^	575.71 ± 9.06^c^
Muscat Ottonel	white	631.38 ± 21.0^c^	541.27 ± 32.3^d^
Timpuriu de Cluj	white	331.34 ± 3.52^f^	380.62 ± 23.5^f^
Napoca	blue-black	1341.37 ± 21.1^a^	1231.38 ± 21.0^a^
Chasselas doré	white	163.26 ± 3.66^h^	148.47 ± 1.69^i^
Muscat Hamburg	blue-black	978.08 ± 12.8^b^	953.04 ± 10.9^b^
LSD 0.05		12.93	19.43

Values are mean ± SD, n = 3; in each column, mean values with different letters are significantly different at P < 0.05.

### Antioxidant activity

#### DPPH - scavenging activity assay

The DPPH· scavenging activity for organic and conventional grapes extracts is presented in Table 3. Significant differences between all the grape samples were obtained, the highest value being recorded for Napoca variety in both culture systems (32.12 ± 1.4 μg Trolox/g for organic and 25.07 ± 1.3 μg Trolox/g for conventional) followed at significant difference by Muscat Hamburg and Muscat Ottonel. The lowest value was obtained for Aromat de Iaşi variety (3.1 ± 0.2 μg Trolox/g organic, 2.1 ± 0.6 μg Trolox/g conventional). Anastasiadi et al. [31] reported DPPH values between 55.7-274.2 μg/g extract in grape skins on four varieties. An interesting study made on conventional and organic grapes in three stages of maturation showed that in stage one of ripening the antioxidant activity was higher for organic grapes (5.70 mM Trolox/g) compared with conventional grapes (4.40 mM Trolox/g [14].

**Table 3:** Antioxidant activity for grape skin using DPPH method

**Variety**	**Colour**	**Organic**	**Conventional**
		**Antioxidant activity**	**Antioxidant activity**
		**(μg Trolox/g sample)**	**(μg Trolox/g sample)**
Aromat de Iaşi	white	3.1 ± 0.2^h^	2.1 ± 0.6^g^
Traminer roz	white	7.3 ± 0.3^g^	5.4 ± 0.24^f^
Riesling italian	white	8.8 ± 0.7^f^	8.1 ± 0.4^e^
Feteasca regală	white	10.1 ± 0.18^e^	12.2 ± 0.9^d^
Muscat Ottonel	white	16.0 ± 0.4^c^	14.4 ± 0.2^c^
Timpuriu de Cluj	white	11.8 ± 1.12^d^	15.73 ± 1.9^c^
Napoca	blue-black	32.12 ± 1.4^a^	25.07 ± 1.3^a^
Chasselas doré	white	7.41 ± 0.3^g^	2.8 ± 0.2^g^
Muscat Hamburg	blue-black	23.01 ± 0.1^b^	22.77 ± 0.5^b^
LSD 0.05		0.86	1.73

Values are mean ± SD, n = 3; in each column, mean values with different letters are significantly different at P < 0.05.

#### ORAC - oxygen radical absorbance capacity

ORAC assay is probably the most widely used hydrogen atom transfer (HAT)-assay which indicates the free-radical scavenging ability of antioxidants against peroxyl radical. The values obtained (Table 4) were significantly different among samples, ranging from 16.8 to 43.5 μmol TE/g in organic grapes and from 14.7 to 40.4 μmol TE/g for conventional ones. These results showed 2.58 (organic) and 2.8 (conventional) - fold differences in antioxidant capacity among genotypes. The highest ORAC values were obtained for blue-black variety Napoca in both farming system (43.5 ± 0.95 μmol TE/g organic; 40.4 ± 0.5 μmol TE/g conventional) while Aromat de Iaşi showed the lowest values (16.8 ± 0.6 μmol TE/g organic; 14.7 ± 1.6 μmol TE/g conventional). The ORAC values obtained were higher than those reported by Cao, Sofic & Prior [32] in red grape samples (7.4 μmol TE/g) and lower than the values reported for 11 grape cultivars from India by Kedage et al. [33] (43.7-46.8 μmol TE/g). ORAC values in red wine grapes ranged from 37.2 mmol TE/kg to 135.8 5 mmol TE/kg in different genotypes [34]. The range of ORAC values obtained in this study was similar with the results of Hogan et al., 2009 [35] for three Virginia grown wine grape extract 22.9-26.7 μmol TE/g.

**Table 4:** Antioxidant activity for grape skin using ORAC method

**Variety**	**Colour**	**Organic**	**Conventional**
		**Antioxidant activity**	**Antioxidant activity**
		**(μmol TE/g sample)**	**(μmol TE/g sample)**
Aromat de Iaşi	white	16.8 ± 0.6^g^	14.7 ± 1.6^g^
Traminer roz	white	17.4 ± 0.2^g^	20.4 ± 1.4^f^
Riesling italian	white	25.6 ± 0.6^e^	25.1 ± 1.4^e^
Feteasca regală	white	28.2 ± 0.9^d^	28.4 ± 1.9^d^
Muscat Ottonel	white	30.1 ± 0.4^c^	32.4 ± 0.2^b^
Timpuriu de Cluj	white	35.8 ± 1.2^b^	30.4 ± 0.9^c^
Napoca	blue-black	43.5 ± 0.9^a^	40.4 ± 0.5^a^
Chasselas doré	white	22.4 ± 1.2^f^	19.8 ± 0.4^f^
Muscat Hamburg	blue-black	25.8 ± 1.8^e^	25.2 ± 1.5^e^
LSD 0.05		0.92	1.14

Values are mean ± SD, n = 3; in each column, mean values with different letters are significantly different at P < 0.05.

### Correlations

**Figure 3:**
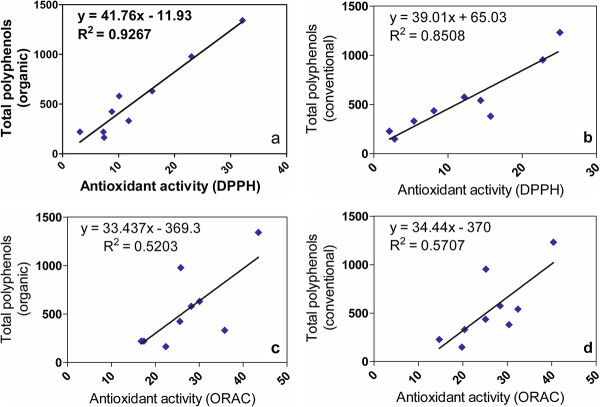
**Linear simple regression analysis.** Correlations between: **a**- DPPH and TPC for organic; **b**- DPPH and TPC for conventional; **c**- ORAC and TPC for organic; **d**- ORAC and TPC for conventional. (TPC: total phenolics content; DPPH: scavenging activity assay; ORAC: oxygen radical absorbance activity).

The relation between the polyphenol content and the antioxidant capacity was measured using different linear correlations. When the total polyphenol content was correlated with DPPH assay the coefficient value was high in both culture systems: *R*^*2*^ = 0.926 for organic (Figure 3a) and *R*^*2*^ = 0.850 for conventional system (Figure 3b). The high regression coefficients values obtained showed a strong correlation between antioxidant capacity and total polyphenol content [31].

The regression coefficient values obtained for total polyphenol content and ORAC assay was lower compared with DPPH assay, but significant, in both systems, conventional (*R*^*2*^ = 0.570) and organic (*R*^*2*^ = 0.520) (Figure 3, c and d).

## Conclusions

In the literature exists just one study regarding the antioxidant activity in organic and conventional grapes (red cultivar Monastrell-*Vitis vinifera*) [14]. Five wine and four table grape varieties cultivated in organic and conventional systems were compared for the carotenoids, total polyphenols content and antioxidant activity. The main carotenoids identified were lutein and ß-carotene. Muscat Ottonel variety has the highest ß-carotene content in both organic and conventional systems and Feteasca regală variety has the highest lutein content. The blue-black cultivars are characterized by high TPC and also high antioxidant activity (except for Muscat Hamburg-ORAC). On the other hand 5 organic grape varieties (Napoca, Muscat Hamburg, Muscat Ottonel, Fetească regală, Chasselas doré) recorded higher TPC compared with conventional and the most organic variants showed a higher antioxidant activity than conventional ones. The pytochemical concentration and antioxidant activity was higher in organically produced tomato juices compared with conventionally ones [36]. Also, apples grown in organic system showed significant differences in chlorogenic acid, flavanols and flavonols compared with conventional grown apples [37].The phenolic compound composition in grapes can be different depending on variety, season and environmental factors [38,39]. Organic agriculture does not use synthetic pesticides and fertilizers and plants being more susceptible to the pathogens action produce higher amount of phenolic compounds [40]. *Vitis vinifera* grape skin (from wine or table grape varieties) can be used as a potential source of natural antioxidants.

### Experimental

#### Biological material and cultivation system

Five wine grape varieties (Aromat de Iaşi, Traminer roz, Riesling Italian, Fetească regală, Muscat Ottonel - whites) and four table grape varieties (Timpuriu de Cluj and Chasselas doré - whites, Napoca and Muscat Hamburg – blue-black) were tested in 2010, Cluj county, Romania, under two types of cultural practices: organic and conventional. Types of culture were differentiated by treatment for diseases, especially downy mildew of grapevine, caused by *Plasmopara viticola* (Berk. & Curt.) Berl. & de Toni which is one of the most serious diseases of grapevine worldwide [41] and by different fertilizing for organic practices and for the conventional. In the conventional system were used chemical fungicides: Ridomil Gold MZ 68 WP (metalaxyl-M 4% + mancozeb 64%), Melody Duo 66.8 WP (iprovalicarb 5.5% + propineb 61.3%), Curzate manox SC (cymoxanil 5% + copper 25% + mancozeb 18%), Quadris max SC (azoxystrobin 22.9%), Folpet 50 WP (folpet 50%) and Dithane M 45 (mancozeb 80%). For the organic treatments were applied ecological products: bordeaux mixture 0.5% + spraying with purine of greater nettle (*Urtica dioica* L.) fermented 1/20 dilution, copper sulphate 1%, Kocide 101 WP (copper hydroxide + metallic copper 50%), bordeaux mixture 1%, soluble sulphur 0.4% and biocontrol agent *Trichoderma harzianum*.

### Grape samples

All samples of grapes were harvested 15 days before technological maturity from the vines grown at the experimental vineyards in Cluj county, Romania. The average yearly precipitation is 660 mm and the vineyards soil type was haplic luvisols. The research planting was organized in the 2.0 m × 1.1 m distance between rows and plants. All the vines were grafted on the same rootstock Selection Openheim nr.4 (SO-4) (*V. berlandieri x V. riparia* rootstock).

Grape berries were taken randomly from different parts of grape vines and different parts of clusters for each sample analyzed. Were collected every 100 berries (in three replicates) for each variety tested in organic and conventional systems. Grape berries were placed in polyethylene bags, transported under refrigerated conditions to the Department of Biochemistry from U. A. S.V. M. Cluj-Napoca within 1,5 h and processed in the same day into laboratory. The skins were manually separated from pulp, weighed and frozen at −20°C until analyzed.

Choosing neighboring plots give us the possibility to compare organic and conventional vineyards in the same soil and climate conditions. In the same spot there were placed two experimental plots: Plot 1-organic cultured and Plot 2-conventionally cultured.

### Chemicals

Methanol, ethyl acetate, petroleum ether, diethyl ether, triethylamine, sodium chloride, anhydrous sodium sulphates were purchased from Sigma Chemical Co. (Madrid, Spain). The purity of carotenoid standards, ß-carotene and lutein, was estimated by registering their UV–Vis spectra and by an individual HPLC run. The ß-carotene and lutein were found to be 96% and 97.5% pure, respectively. All other chemicals were purchased from Merck (Darmstadt, Germany).

### Carotenoids extraction and separation

Total carotenoids were extracted from 20 g fresh grape skin using a mixture of methanol/ethyl acetate/petroleum ether (1:1:1, v/v/v). After filtering the extract, the residue was re-extracted twice with the same mixture of solvents, following the procedure described by Breithaupt and Schwack [42]. The extracts were combined and partitioned in a separation funnel, successively with water, diethyl ether and saturated saline solution. The ether phase was evaporated to dryness under vacuum, using a rotary evaporator at 35°C. The samples were kept under nitrogen, at −20°C until further utilization. HPLC analyses for individual carotenoids were carried out on Shimadzu LC20 AT controller system with PDA detector SPD-M20A, using a reversed phase Hibar 250–4 Lichrosorb C18 column (250 × 4.6 mm), 5 μm. A dual gradient mobile phase was used and made of acetonitrile: water (9:1, v/v), with 0.25% triethylamine (solvent A) and ethyl acetate with 0.25% triethylamine (solvent B). The gradient started with 15% B to 50% B from 0 to 16 min and continued isocratically up to 30 min. The flow rate was 1 ml/min. All chromatograms were monitored at 450 nm. The HPLC peaks were identified by using parallel HPLC runs with carotenoid standards as well as by recording the UV–Vis spectra and their comparison with known carotenoid spectra [43]. Finally the results were expressed as micrograms carotenoid/kg FW (μg/kg sample).

### Polyphenols extraction

For polyphenols extraction 10 g of grape skin, in three replicated each, was extracted by grinding the sample 1 min at 20,000 rpm in a blender (Ultra-Turrax Miccra D-9 KT Digitronic, Germany) with 10 ml of acidified methanol (85:15 v/v, MeOH:HCl). The homogenate was centrifuged at 3500 rpm for 10 min. The extract was separated and the residual tissue was re-extracted until the extraction solvents became colorless. The filtrates were combined in a total extract and dried using rotary evaporator at 40°C.

### Total polyphenols

The amount of total polyphenols in grape skins was determined using modified Folin-Ciocâlteu colorimetric method [44]. Grape skin extracts (25 μl each) were dissolved in methanol and further dilution were performed to obtain readings within the standard curve made with gallic acid (R = 0.997). The extracts were oxidized by Folin-Ciocâlteu reagent (120 μl) and after 5 min, were added 340 μl Na_2_CO_3_ for neutralization. The samples were kept 90 min in the dark followed by the reading of the absorbance at 750 nm. The results were expressed as milligram of gallic acid/1 kg sample (mg GAE/1 kg sample).

### Antioxidant activity

#### DPPH - scavenging activity assay

The DPPH scavenging activity assay was done according to a method reported by Brand-Williams, Cuvelier and Berset [45]. 80 μM of DPPH· solution was freshly prepared in 95% methanol. 250 μl of DPPH· solution was allowed to react with 35 μl sample and the absorbance was measured at 515 nm, for 60 min. The chemical kinetics of the extracts was recorded. The antioxidant activity was calculated as follows:

% DPPH· scavenging activity = (1-[A_sample_/A_control t=o_]) 100 (1)

The results were expressed as microgram Trolox/g sample (μg TE/g sample).

#### ORAC - oxygen radical absorbance activity

The oxygen radical absorbance capacity (ORAC) measure the peroxyl radical scavenging activity using as standard 6-hydroxy-2,5,7,8-tetrametylchroman-2-carboxylic acid (Trolox) [46]. A fluorescein stock solution (4x10^-3^ μM) was made in phosphate buffer (75 mM, pH 7.4) and kept in the dark at 4°C. Before utilization, the fluorescein stock solution was diluted with the phosphate buffer. The fluorescein solution was added to each Trolox standard and grape sample (25 μl) made in phosphate buffer and incubated for 30 min, at 37°C. The reaction was initiated by adding 25 μl 2,2^’^-azobis-2-amidinopropane (AAPH) and the fluorescence was measured kinetically at excitation wavelength 485 nm and emission wavelength 535 nm, every minute using a fluorescence microplate reader BioTek (Synergy HT, BioTek Instruments, Winooski, VT). The ORAC values for each grape extract were calculated using the net area under the decay curves and were expressed as micromoles Trolox equivalents/g sample (μmol TE/g sample).

### Statistical analysis

The design of the experiment was linear, bifactorial type (type of culture x cultivar). The data were expressed as mean ± standard deviation (SD) from three replicates for each sample. In order to determine the significant differences between values, analysis of variance (ANOVA) and Duncan multiple range tests (MRT) were performed. Significance of difference was defined at the 5% level (p < 0.05).

## Abbreviations

TPC, Total phenolics content; DPPH, Scavenging activity assay; ORAC, Oxygen radical absorbance capacity; HAT, Hydrogen atom transfer; HPLC, High performance liquid chromatography; FW, Fresh weight; U. A. S.V. M., University of Agricultural Sciences and Veterinary Medicine.

## Competing interests

The authors declare that they have no competing interests.

## Authors’ contributions

CIB carried out the experimental design, analyses, interpretation of results and preparation of the paper. NP and ACB, contributed equally to the collection of data and development of the grapes sampling. CM and FD* contributed equally to the extraction procedure for carotenoids and polyphenol analysis, statistical analysis and preparation of the paper. AB contributed to the separation, identification and quantification of carotenoids and measuring the antioxidant activity. All authors read and approved the final form of the manuscript.
